# The role of functional and structural interhemispheric auditory connectivity for language lateralization - A combined EEG and DTI study

**DOI:** 10.1038/s41598-018-33586-6

**Published:** 2018-10-18

**Authors:** Saskia Steinmann, Rom Amselberg, Bastian Cheng, Götz Thomalla, Andreas K. Engel, Gregor Leicht, Christoph Mulert

**Affiliations:** 1 Psychiatry Neuroimaging Branch, Department of Psychiatry and Psychotherapy, University Medical Center Hamburg-Eppendorf, Hamburg, Germany; 2 Department of Neurology, University Medical Center Hamburg- Eppendorf, 20246 Hamburg, Germany; 3 Department of Neurophysiology and Pathophysiology, University Medical Center Hamburg-Eppendorf, 20246 Hamburg, Germany; 4 Centre for Psychiatry and Psychotherapy, Justus-Liebig-University, Giessen, Germany

## Abstract

Interhemispheric connectivity between auditory areas is highly relevant for normal auditory perception and alterations are a major factor for the development of auditory verbal hallucinations. Surprisingly, there is no combined EEG-DTI study directly addressing the role of functional and structural connectivity in the same group of subjects. Accordingly, nothing is known about the relationship between functional connectivity such as gamma-band synchrony, structural integrity of the interhemispheric auditory pathways (IAPs) and language lateralization as well as whether the gamma-band synchrony is configured on the backbone of IAPs. By applying multimodal imaging of 64-channel EEG and DTI tractography, we investigated in 27 healthy volunteers the functional gamma-band synchrony between either bilateral primary or secondary auditory cortices from eLORETA source-estimation during dichotic listening, as well as the correspondent IAPs from which fractional anisotropy (FA) values were extracted. Correlation and regression analyses revealed highest values for gamma-band synchrony, followed by FA for secondary auditory cortices, which were both significantly related to a reduced language lateralization. There was no such association between the white-matter microstructure and gamma-band synchrony, suggesting that structural connectivity might also be relevant for other (minor) aspects of information transfer in addition to gamma-band synchrony, which are not detected in the present coupling analyses. The combination of multimodal EEG-DTI imaging provides converging evidence of neural correlates by showing that both stronger pathways and increased gamma-band synchrony within one cohort of subjects are related to a reduced leftward-lateralization for language.

## Introduction

Conscious auditory perception relies on a timely interhemispheric interaction between right and left auditory cortices^1^. Gamma-band oscillations and their synchronization in neural networks has been proposed as a fundamental mechanism that subserves such precise temporal integration to enable a coherent perception^2–6^. Moreover, previous data highlight that these distributed neuronal interactions are dynamically configured on the base of anatomical pathways^7–9^. Alterations of both, the connectome and gamma rhythmic activity, are suggested by the interhemispheric miscommunication theory^10^ to constitute a pathophysiological mechanism underlying altered perception as it is observed, for instance, in schizophrenia patients^11^ with auditory verbal hallucinations (AVH)^12–15^.

Much work has been devoted to the role of the interhemispheric gamma-band coupling^1^ and the structure of the interhemispheric auditory pathways (IAPs)^16^ for auditory perception using the dichotic listening task. The dichotic listening task is the most frequently applied paradigm for examining language lateralization^17^. Here, two different consonant-vowel syllables (e.g. “ba”-“ga”) are presented simultaneously to the right (RE) and to the left ear (LE) and the listener is required to report the syllable they heard best. Due to the left-hemispheric specialization for language and the predominance of the contralateral fibers linking the RE directly with the left-dominant processing areas^18^, healthy right-handers more often report syllables presented to the RE, a phenomenon known as the right ear advantage (REA)^19^. In contrast, the LE syllable initially enters the right auditory cortex and requires further transport through IAPs to be processed in the speech-dominant hemisphere^20^. Using functional and effective EEG-based connectivity analysis, it has been shown that this causal interhemispheric transfer of auditory information during LE performance runs from the right to the left posterior parts of the superior temporal gyrus (pSTG)^21^, and further, is mediated by synchronized gamma-band oscillations^1^. In line with that, a previous DTI tractography study has shown that a larger midsagittal size of the posterior part of the corpus callosum – which is considered as the callosal area (i.e. splenium) where the IAPs cross^22^ – was also positively correlated with conscious perception of LE syllables, whereas the RE reports showed no such association^16^. Furthermore, this study revealed a huge interindividual variability in callosal topography linking homolog pSTG, emphasizing the functional relevance of interindividual white-matter differences. However, these findings indicate that stronger IAPs as well as increased gamma-band synchronization between homolog pSTG are both contributing to conscious perception of LE syllables.

First evidence of a mutual interaction of functional and structural connectivity was provided by Engel *et al*.^9^, demonstrating that the interhemispheric gamma-band synchronization between homolog visual cortices of cats was dependent on transcallosal pathways and abolished after cut-off of the corpus callosum. So far, no multimodal approach exists that combines measures of DTI with EEG-based gamma-band functional connectivity pattern between bilateral auditory cortices and with dichotic listening performance, although it appears likely that interhemispheric gamma-band synchrony is configured on the backbone of IAPs. Such an approach could help to provide a better understanding of how coupling of EEG-rhythms depends on the white-matter microstructure, and what functional and structural interhemispheric aspects finally drive auditory perception. DTI represents a MRI-based, noninvasive technique that measures the diffusion of water molecules in biological tissue to infer the white-matter connectivity of the brain^23–25^. To date, fractional anisotropy (FA) is the most frequently used DTI measure and is assumed to reflect white-matter fiber organization and integrity^26^.

Thus, the present study examines, in particular, the association between language lateralization (measured with the dichotic listening task) and (1) functional gamma-band synchronization (derived from EEG recording conducted during dichotic listening task), as well as (2) with the structural DTI information of the IAPs connecting either bilateral Heschl’s gyri or pSTG. It was hypothesized that a reduced left lateralization for language is related to (1) structural integrity measured by FA and, (2) increased functional gamma-band synchrony between homolog auditory cortices, and further, that (3) the magnitude of the interhemispheric gamma-band synchrony is related to structure of the IAPs.

## Material and Methods

### Participants

Thirty healthy, right-handed German native speakers (15 male) aged between 20–50 years were recruited, but twenty-seven were finally involved into the analyses. Three volunteers dropped out due to hearing problems or technical artifacts. Exclusion criteria were left-handedness, bi-manual-handedness, any past or current psychiatric or neurological disorder (including substance use disorders), a history of schizophrenia up to second degree relative, a hearing disorder or serious medical condition, bilingually or polyglott, and an IQ lower than 70. Moreover, the usual exclusion criteria for MRI studies (such as presence of metal parts or devices sensitive to magnetic fields) or claustrophobia was applied. The participants’ handedness was verified with the empirically validated Edinburgh Handedness Inventory^27^. To ensure normal hearing in both ears, all participants were screened with pure tone audiometry for frequencies between 125 and 8000 Hz (Esser Home Audiometer 2.0). Participants with an auditory threshold higher than 25 dB, or an interaural difference larger than 15 dB in any of the frequencies were excluded from the study. This study was approved by the local ethics commission of the Medical Association Hamburg; all applied methods were in accordance with all relevant guidelines and regulations. After participants received a complete description of the experimental procedures, written informed consent according to the Declaration of Helsinki was obtained. Demographic data for all participants are presented in Table 1.

**Table 1:** Demographic, behavioral and neuroimaging characteristics. FA: fractional anisotropy; LPS: lagged phase synchronization; pSTG: posterior superior temporal gyrus.

All participants (n = 27)		
Variable	Mean ± SD	Range
Age (years)	30.19 ± 8.54	20–50
Sex (M/F)	14/13	
Handedness	90.62 ± 13.89	40–100
Education (years)	17.0 ± 1.93	13–21
Right ear reports (%)	134.04 ± 28.29 (55.9%)	85–184
Left ear reports (%)	81.26 ± 17.18 (33.8%)	50–112
Error reports (%)	24.81 ± 15.42 (10.3%)	2–55
Laterality Index (LI)	23.56 ± 19.70	(−11)–57
Response time LE	2.99 ± 30.95 sec	2.15–3.49 sec
Response time RE	2.89 ± 26.85 sec	2.15–3.39 sec
Verbal intelligence	109.96 ± 8.84	86–133
FA of Heschl’s gyrus	0.45 ± 0.027	0.38–0.49
FA of pSTG	0.44 ± 0.020	0.41–0.49
LPS of Heschl’s gyrus	0.0105 ± 0.0021	0.01–0.02
LPS of pSTG	0.0111 ± 0.0025	0.01–0.02

### Paradigm

**Figure 1:**
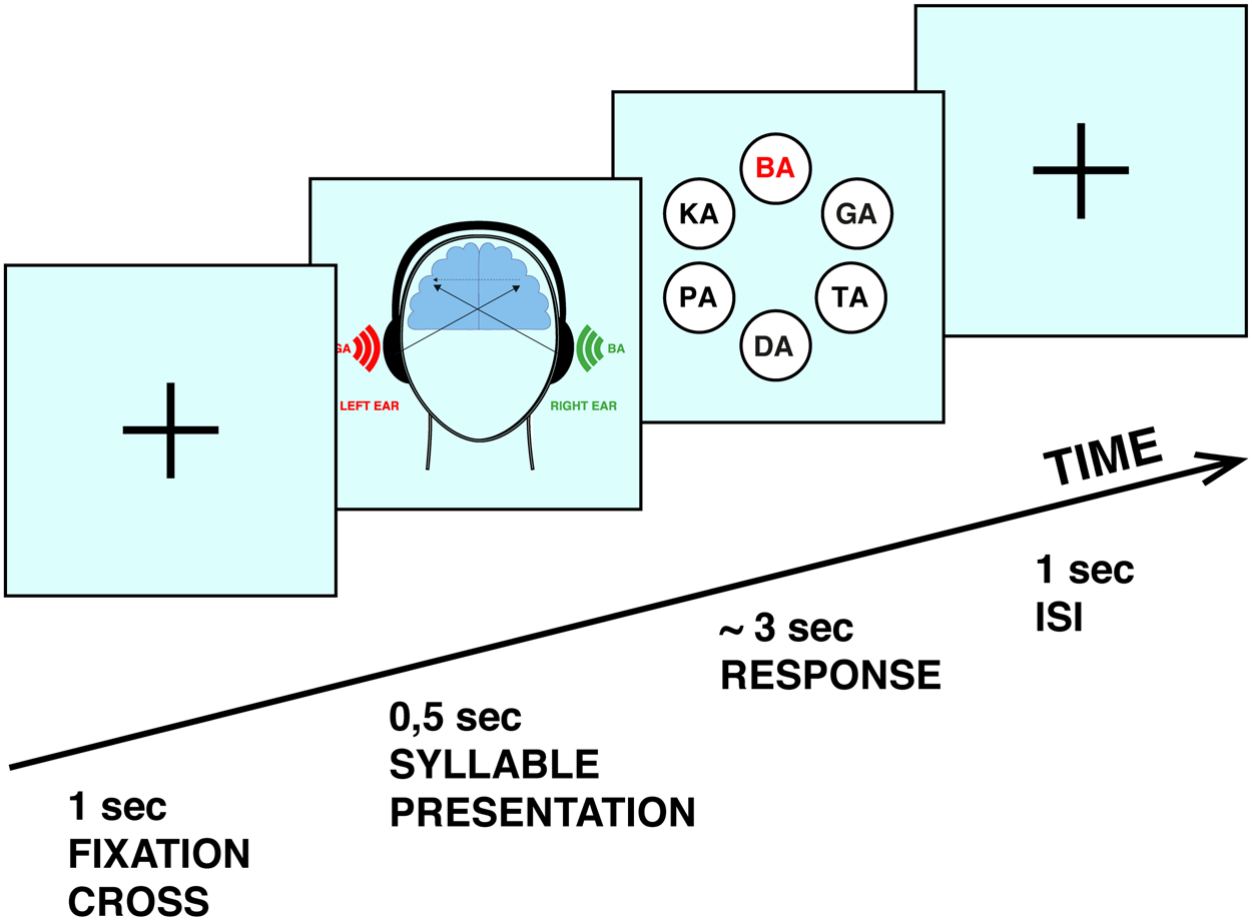
Dichotic listening paradigm. After a fixation cross presented on a blank screen for 1000 ms, the syllables were presented bilaterally. Next, the screen showed all six syllables presented in a circular formation. By clicking with the right (dominant) hand the left mouse button it was possible to navigate through the six answer alternatives and with the right mouse button the selection was confirmed. Between the offset of the visual presentation and the onset of the next auditory stimulus a stable interstimulus interval of 1000 ms was applied.

The dichotic listening task used was the same as in our previous studies^1,15,21^. In brief, six different consonant-vocal syllables (/ba/, /da/, /ka/, /ga/, /pa/, /ta/) were paired and presented simultaneously: one syllable to each ear, resulting in 12 different combinations as only syllables with the same voice-onset time were combined^28^. Participants were instructed to report after each trial which syllable was perceived best. The response was given via button press with the right-dominant hand, while a response screen, which appeared right after hearing the syllable pair, showed all six syllables presented in a circular formation (see Fig. 1). For further analyses a behavioral laterality index (LI) was calculated for every subject according to the formula:

$${\rm{LI}}=[100\ast ({\rm{RE}}-{\rm{LE}})/({\rm{RE}}+{\rm{LE}})],$$

where RE = number of right ear reports and LE = number of left ear reports. The laterality index is a scaling factor that varies continuously from −100 to 100 with a LI >0 for the typical left-hemispheric dominance of language, or a LI <0 for right-hemispheric dominance.

### EEG recording

The recording took place in a sound-proof and electrically shielded cabin, while participants listened through closed system headphones (Sennheiser, HAD 200) to the randomly presented 240 syllable pairs at approximately 75 dB. The EEG recordings were conducted with 64 Ag/AgCl electrodes mounted on an elastic cap (ActiCap, Brain Products, Munich, Germany), including four EOG channels to monitor eye movements, using the Brain Vision Recorder 1.10 (Brain Products, Munich, Germany). Data were recorded and digitized at a sampling rate of 1000 Hz. Impedances were kept below 5 kΩ. Offline processing was carried out using Brain Vision Analyzer 2.0 (Brain Products, Munich, Germany). The data was Butterworth zero phase bandpass filtered from 20 to 120 Hz (IIR, 12 dB/octave) and down-sampled to 500 Hz. All channels were re-referenced to common average and FCz was recovered as a regular channel. Epochs with muscle artifacts in any channel were identified by visual inspection and rejected from further analysis. Independent component analysis (ICA) was applied to identify and remove blink, horizontal eye movements, electrocardiographic, muscle and saccadic spike potential artifacts based on their characteristic topographies, time-courses, and frequency distributions^29^. To control for saccadic spike potential artifacts in the gamma frequency range^30^ an additional “radial electro-oculogram channel” (REOG) was derived following the procedure described by Keren *et al*.^31^. Subsequently, the artifact-free data was segmented in epochs ranging from −200 to 1848 ms regarding the stimulus onset, and all correct-response epochs were exported for further analysis in the source-space.

### Functional gamma-band connectivity analysis

**Figure 2:**
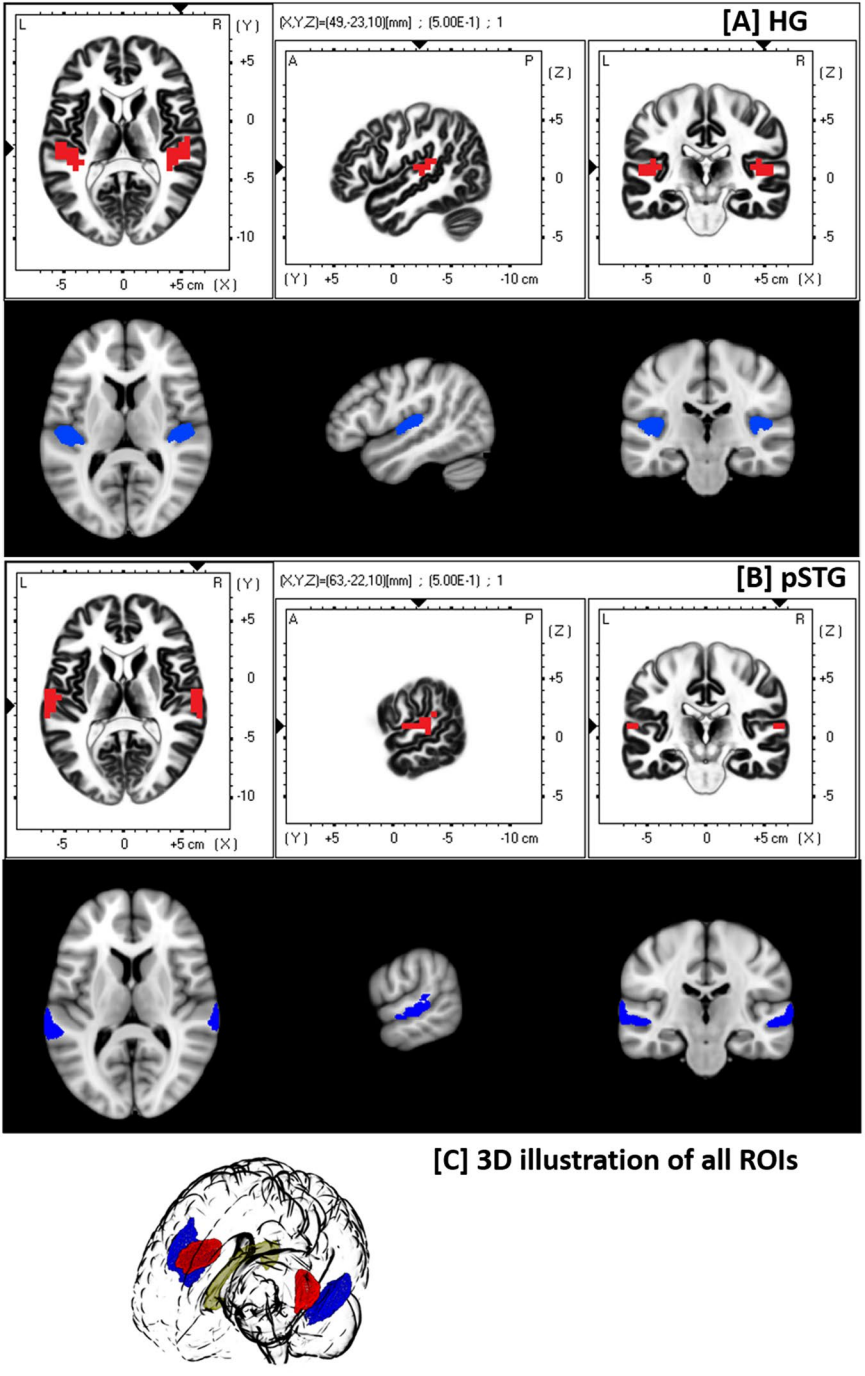
White background (red): EEG-ROIs created in LORETA in standard space. Black background: DTI-ROIs created in FSL in standard space (blue). (**A**) Heschl’s gyrus (**B**) posterior superior temporal gyrus (pSTG) (**C**) 3D illustration of Heschl’s gyri (red), pSTG (blue) and midsagittal callosal body (yellow).

Similar to our previous studies^1,21,32^, all further analyses were executed with the LORETA KEY software package^33^ investigating functional connectivity computed as “lagged phase synchronization” (LPS)^34^. LPS was assessed within two a priori defined regions-of-interests (ROIs): one candidate area for the processing of any type of sound is the right and left Heschl’s gyrus (BA41), and another one is pSTG (BA42) which has been related to the processing of complex sounds and speech sounds^35,36^. The ROIs were defined using the anatomical definitions provided by the eLORETA software based on the Talairach Daemon (see Fig. 2, white row). Based on our previous dichotic listening work, where we did not find LPS differences in any of the other frequencies (delta, theta, alpha, beta) as well as an effect in all gamma subbands (30–50 Hz, 50–90 Hz), analysis was focused on the whole gamma-band range (30–100 Hz) in all correct right and left ear responded trials (i.e. bilateral). LPS across all the voxels included in the ROIs was based on the average of all LPS values, which were calculated for the connectivity between every voxel of ROI A and every voxel of ROI B. For all single trials, time-varying frequency analysis was done using a short time Fourier transform (sliding Bartlett-Hann window function) with a window width of 100 ms. LPS was calculated using cross spectra derived from that transformation. In a time frame from 0 to 800 ms after stimulus presentation results were extracted every 100 ms and averaged to one final mean value. Details of EEG electrode placement, the computation methods and its advantages have been provided previously^1,32^.

### Diffusion Tensor Imaging (DTI)

We performed MRI at 3T field strength using a Magnetom TIM Trio (Siemens, Erlangen, Germany) equipped with a gradient system providing a maximum strength of 40 mT/m using a 12-channel head coil. For structural imaging, a high-resolution 3D T1-weighted magnetization-prepared rapid gradient echo (MPRAGE) data set was acquired using the following sequence: TE/TR = 2.98/2300 ms, TI 1100 ms, flip angle 9°, 256 × 192 × 240 matrix with FOV 256 × 192 × 240 mm^3^ with an acquisition time of 7 min 23 sec. Heads of participants were stabilized using foam pads to minimize movement artifacts. DTI data was measured with echo planar imaging (EPI) covering the whole brain. The protocol parameter consists of TE/TR = 86/7700 ms, bandwidth = 1502 Hz/Px, 104 × 128 matrix, 64 axial slices of 2 mm without inter-slice gap, resulting in an isotropic voxel size of 2.0 × 2.0 × 2.0 mm^3^. Gradient pulses along 60 different directions derived with a b-value of 1000 s/mm^2^. Non-diffusion weighted images (b-value 0 s/mm^2^) were acquired after every tenth image to guide registration of individual diffusion images. To minimize the sensitivity against frequency drifts, the protocol was split into three acquisitions yielding a total measurement time of 9 min 39 sec. All images were visually inspected for absence of motion and ghosting artifacts.

### DTI regions-of-interest (ROI) definitions

Five ROIs were extracted from a brain template in MNI152 standard space, which were transformed into individual diffusion space with the following steps: First, the individual T1 weighted MRI images (sMRI) were registered to the DTI image using FMRIB Software Library tool’s (FSL 5.0, Smith *et al*. 2004) Linear Image Registration Tool (FLIRT). The co-registration between standard space and sMRI, as well as between standard space and DTI images were carried out using FSL’s Non-linear Image Registration Tool (FNIRT; i.e. nonlinear transformation of initial affine 12 degrees of freedom registration). Five ROIs were defined using FSL’s Atlas tool: [1] right Heschl’s gyrus, [2] left Heschl’s gyrus, [3] right pSTG, [4] left pSTG, and [5] midsagittal mask of the corpus callosum. The ROIs have been chosen in accordance with the ones defined by eLORTA for the EEG analysis (see Fig. 2). Heschl’s gyrus and pSTG ROIs were defined by the “Harvard Oxford cortical structural atlas”^37^, and the corpus callosum mask by the “Juelich Histological Atlas”; all with a probability threshold of 20%. Overlapping voxels appearing in both Heschl’s gyri and pSTG have been removed from pSTG. ROIs 1 to 4 were then registered into structural space (sMRI) of each subject and restricted to cortex by multiplication with a binarized cortex image created by the FAST tool of FSL. Finally, the ROIs were transformed into diffusion space of each subject. Corpus callosum-ROI was registered directly into diffusion space of each subject while considering only the midsagittal cut of the ROI (x = 64 out of 127).

### DTI pre-processing and probabilistic tractography

DTI DICOM volumes were converted to NIfTI format and pre-processed using FSL. After brain extraction, motion and eddy current correction of the diffusion weighted images, the FMRIB’s Diffusion Toolbox (FDT) was used for tensor fitting. Next, BEDPOSTX (Bayesian Estimation of Diffusion Parameters Obtained using Sampling Techniques) function was applied to estimate fiber orientation in each voxel. The FDT’s PROBTRACKX command was then used to initiate four probabilistic tractographies of the respective ROIs with the following parameters: step length = 0.5 mm; curvature threshold = 0.2. To ensure that only the interhemispheric auditory pathways (IAPs) were tracked, tractography had to pass through the corpus callosum mask (waypoint) and was conducted for each hemisphere twice; i.e., using, for instance, the right Heschl’s gyrus as seed mask and the left Heschl’s gyrus as termination mask, and vice versa. Furthermore, each tract was normalized (intensity divided by waytotal) and a probability threshold of 1% was set to reject unlikely fibers. The overlapping tracts pairs (Heschl’s gyrus R > L & Heschl’s gyrus L > R, pSTG R > L & pSTG L > R) were then combined into two maps (Heschl’s gyrus, pSTG), binarized, and multiplied with the individual FA map. Finally, mean value of FA within the final tracts of Heschl’s gyri and pSTG was calculated. Although the focus was on FA-value, axial- (AD), radial- (RD), and mean-diffusivity (MD) was also extracted to look for potential pathway properties that could predict dichotic listening performance.

### Statistics

Data analysis was performed using SPSS version 24. Since it is known that age^38,39^ and gender^40^ influence FA values, an ANCOVA with age as covariate was used to compare FA values between male and female. To assess the association between white-matter microstructure, functional gamma-band synchrony and auditory perception, one-tailed Bonferroni-holm corrected correlations based on the strong a priori hypothesis was performed between means of FA, LPS (of Heschl’s gyrus and pSTG) and dichotic listening performance (LI, number of LE- or RE-reports) using either Spearman’s or Pearson’s correlation coefficient (p < 0.05). Bootstrapping was used to calculate 95% confidence intervals (CI) around each significant correlation.

Next, a combined multiple-regression analysis using the backward elimination procedure with LI as dependant variable and FA, MD, AD, RD, and LPS as independent variables was performed to examine the extent to which the pathway properties and the gamma-band synchrony of pSTG can predict LI.

## Results

### Dichotic listening task performance

Across all participants 55.9% of trials were RE reports and 33.8% were LE reports, indicating the typical REA with mean LI of 23.56 (SD = 19.70). Errors (i.e. reporting a syllable that was not presented) occurred in 10.3% of the trials. 23 out of 27 participants showed a positive LI, whereas 4 participants had a negative LI. In addition, there was the typical significant difference in reaction times (t = 3.543, p = 0.002) with faster answers for RE (mean: 2.89 sec) than LE reports (mean: 2.99 sec).

### DTI fiber tracking

**Figure 3:**
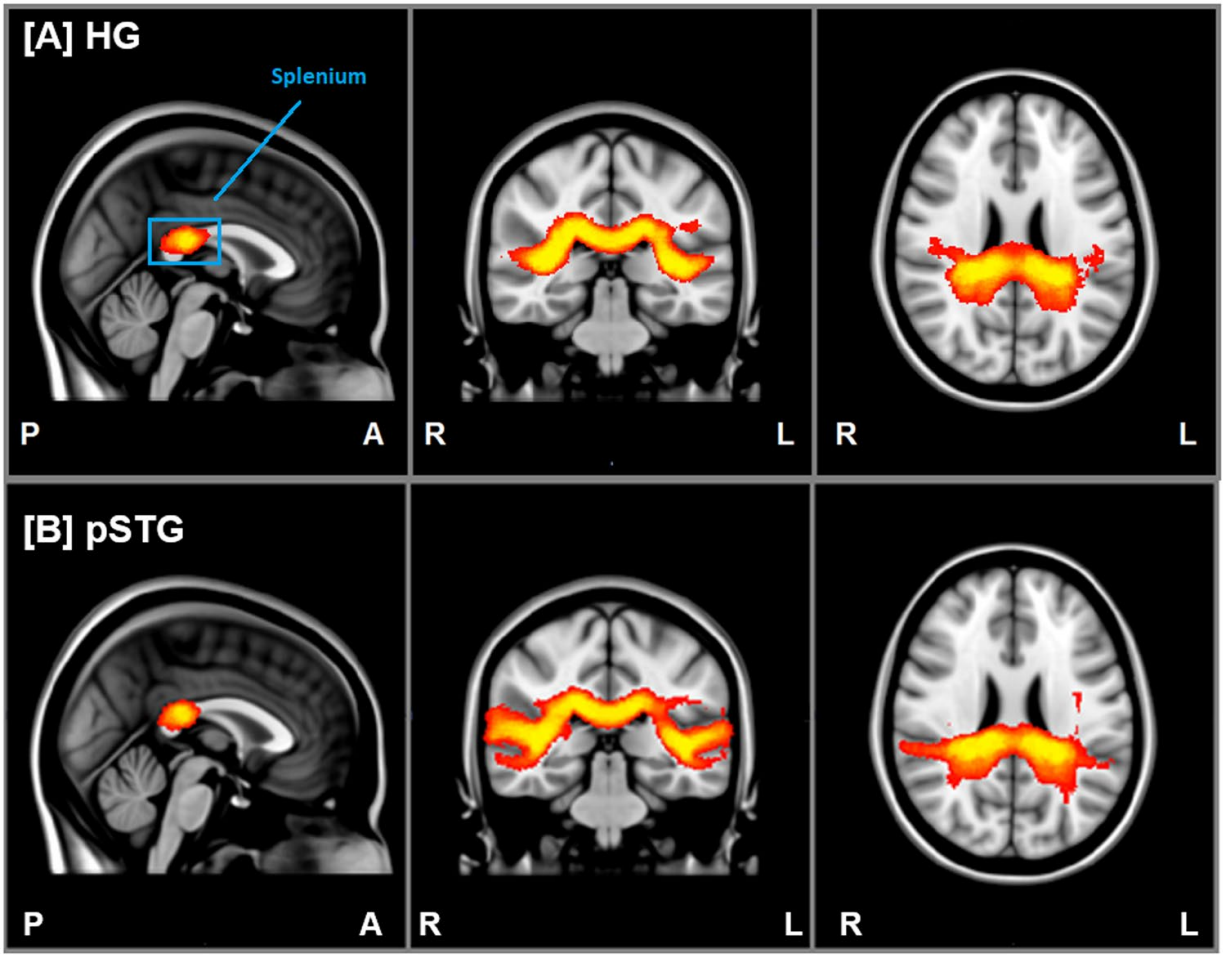
Group mean tractographies. Cumulated fiber densities per voxel connecting (**A**) Heschl’s gyrus (HG) and (**B**) posterior superior temporal gyrus (pSTG) on both hemispheres in standard space (MNI152). Note that the intensity (red-yellow gradient) represents how many tracks pass through each voxel. P: posterior; A: anterior; R: right; L: left.

The results of the fiber tracking are shown on a standard MNI152-templated in Fig. 3, verifying that the IAPs interconnecting Heschl’s gyri or pSTG on both hemispheres cross within the posterior fifth of the corpus callosum (splenium). The ANCOVA with age as a covariate revealed no significant differences of FA-values between male and female (p > 0.12).

### Association between FA and dichotic listening performance

**Figure 4:**
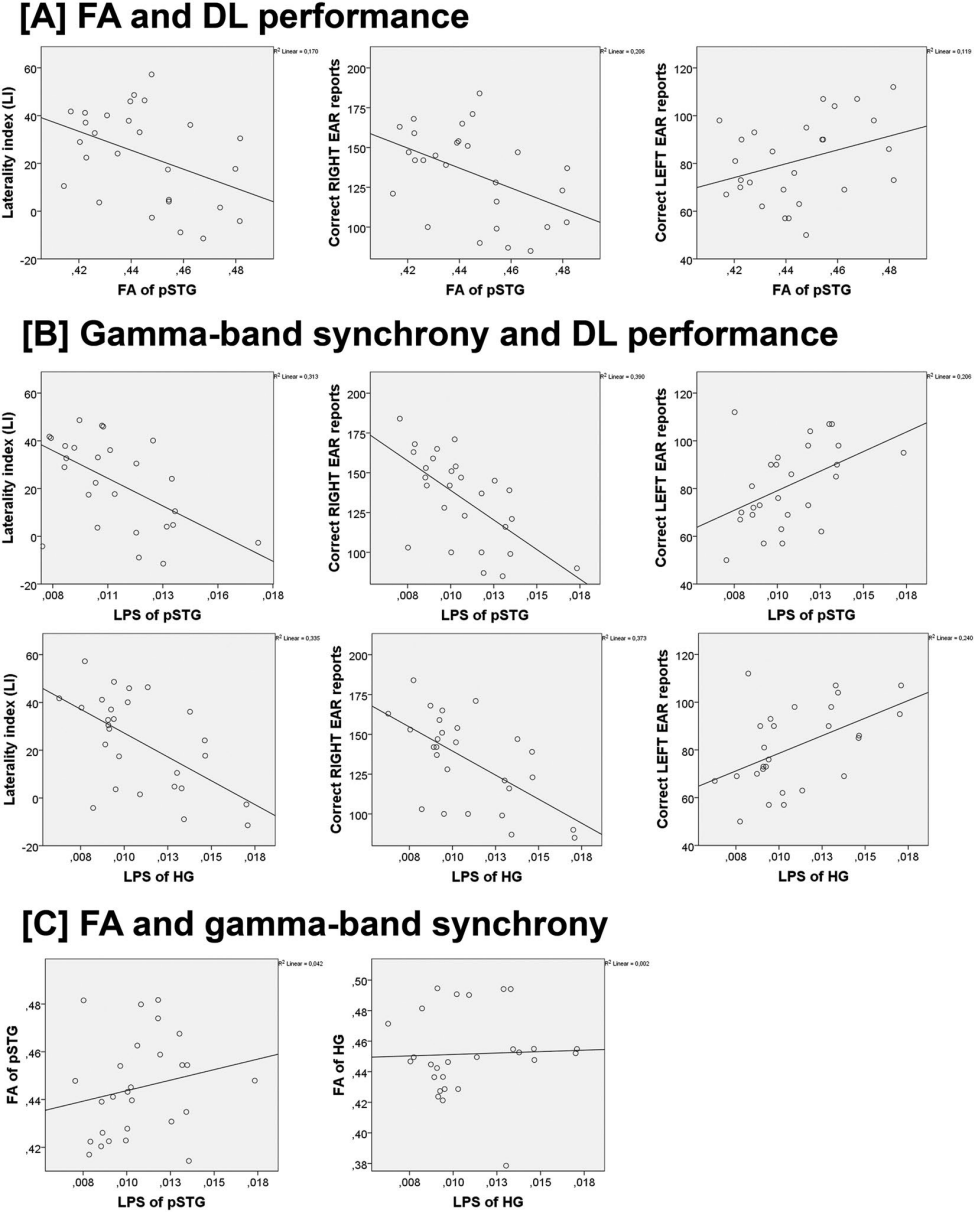
Scatterplots showing the significant association between (**A**) the mean fractional anisotropy (FA) value of the interhemispheric auditory pathway connecting homolog posterior superior temporal gyrus (pSTG), or (**B**) the mean gamma-band synchrony (lagged phase synchronization/LPS) connecting either pSTG or Heschl’s gyrus (HG), and the dichotic listening (DL) performance (all p < 0.05). (**C**) Scatterplots showing the non-significant relation between gamma-band synchrony and the mean FA values of the interhemispheric auditory pathway connecting either homolog pSTG or HG.

Relating the dichotic listening performance to the results of the probabilistic tractography, a negative correlation was observed between FA of pSTG tract and LI (r = −0.412, Bonferroni-holm corrected p = 0.032, bootstrap CI = −0.657 to −0.088). When analyzing correct number of LE and RE reports separately, a significant positive correlation was found between pSTG-based tracked FA and the LE reports (r = 0.345, Bonferroni-holm corrected p = 0.039, bootstrap CI = 0.041 to 0.628, while a negative correlation was found with RE reports (r = −0.454, Bonferroni-holm corrected p = 0.027, bootstrap CI = −0.687 to −0.157, see Fig. 4A). No such association was found between Heschl’s gyrus based tracked FA values (p > 0.10).

### Association between gamma-band synchrony and dichotic listening performance

Interhemispheric LPS between homolog pSTG was negatively correlated with LI (r = −0.559, Bonferroni-holm corrected p = 0.006, bootstrap CI = −0.791 to −0.189) and this relationship was also observed with the number of correct RE reports (r = −0.625, Bonferroni-holm corrected p = 0.005; bootstrap CI = −0.827 to −0.285), while a significant positive correlation was found with the number of correct LE reports (r = 0.454, Bonferroni-holm corrected p = 0.018; bootstrap CI = 0.052 to 0.749). LPS between homolog Heschl’s gyrus was negatively correlated with LI (rho = −0.488, Bonferroni-holm corrected p = 0.015, bootstrap CI = −0.754 to −0.109) and also with the number of RE reports (rho = −0.571, Bonferroni-holm corrected p = 0.004, bootstrap CI = –0.794 to −0.233), while the number of LE reports were positively correlated (rho = −0.413, Bonferroni-holm corrected p = 0.016, bootstrap CI = 0.019 to 0.728) (see Fig. 4B).

### Association between DTI measures, gamma-band synchrony, and LI

Although visual inspection hints that increased gamma-band synchrony is associated with higher FA-values of the IAPs linking both pSTG, the statistical analyses failed to reach significance, as it was the same for Heschl’s gyrus (both >  = 0.15) (see Fig. 4C). The multiple-regression analysis (which was focussed on the pSTG in accordance with the previous DTI results), with LI as dependent variable and all DTI measures (i.e. FA, MD, AD, RD) and LPS as independent variables, again revealed that FA (beta = −0.311, t_(24)_ = −1.93, p = 0.065) and LPS (beta = −0.496, t_(24)_ = −3.08, p = 0.005) were the only variables providing a unique contribution to LI prediction (F_(24)_ = 8.177, p = 0.002). Moreover, LPS was a stronger predictor than FA (beta = −0.496 > −0.311), which was marginally below the significant level (p = 0.65). There was also no significant association between gamma-band synchrony and the other DTI parameters of MD, AD, or RD (all p > 0.15).

## Discussion

In this multimodal study, we combined EEG measured functional gamma-band synchrony, DTI probabilistic tractography of the interhemispheric auditory pathway (IAPs) and dichotic listening performance of 27 healthy volunteers to investigate the role of this white-matter pathway in coordinating gamma-band synchrony that is required for conscious auditory perception of left ear (LE) syllables. The data supports two of our main hypotheses: (1) anatomical stronger IAPs and (2) increased gamma-band synchronization between bilateral parts of the superior temporal gyrus (pSTG) were both related to a reduced laterality index (LI). However, there was no significant association between the white-matter structure and functional gamma-band connectivity. To the best of our knowledge, this is the first multimodal study combining DTI and EEG demonstrating that stronger pathways and increased gamma-band synchrony within one cohort of subjects are related to a reduced leftward-lateralization for language.

Behaviourally, we found the characteristic right ear advantage (REA), which is best explained by the structural model^41^, suggesting that the REA emerges due to the left-hemispheric specialization for language and the predominance of the contralateral pathways during simultaneous stimulation. As a consequence, the LE syllable initially enters the right auditory cortex and requires further interhemispheric transport to the speech-dominant hemisphere to be processed and consciously perceived. Our finding of strong association between higher pSTG-based extracted fractional anisotropy (FA) values and reduced LI as well as with higher number of LE reports and a lower number of right ear (RE) reports supports this model and indicates that the IAPs is a contributor to the degree to which language is functionally lateralized. This is in line with a previous functional and structural MRI study demonstrated that a larger midsagittal callosal surface area was associated with greater left-hemispheric activity during language tasks in posterior temporal and inferior frontal brain regions^42^. Notably, Westerhausen exhibited using DTI-based tractography that a greater midsagittal size of the splenium was related to an increased number of LE reports during dichotic listening^16^. While this study compared the number of midsagittal voxels, the current study used an advanced approach of quantifying the longitudinal trajectory of FA. The FA is a pivotal measure of directionality and voxel-to-voxel coherence of MRI-detectable water diffusion in white-matter fibers^43^, and is typically higher in compact fibers than in tissue with an inhomogeneous structure, such as crossing fibers^44,45^ or pathological areas^46,47^. FA is thought to reflect myelin, axon diameter, axon permeability, and packing density^24,48,49^ and renders an estimate of microstructural integrity^26,50^, which in turn is associated with conduction velocity^51^. Based on the role of the corpus callosum in connecting both hemispheres for interaction^52^, our observed correlation suggests that stronger pathways facilitate fast information transfer and integration of processes across the hemispheres and reduce the typical left-lateralization for language. Insights into the functional relevance of the IAPs were provided by clinical studies showing that callosal lesions can result in specific functional impairments of speech processing and spoken language comprehension^53–55^ and significantly reduced LE reports during dichotic listening^20,56,57^. Besides, the DTI-based tractography of this study is congruent with the topographical callosal organization since its projections linking homolog pSTG cross within the splenium^22^ and are in accordance with one prior study that depicted an identical tract^16^.

Moreover, the EEG-based analyses revealed that interhemispheric gamma-band synchrony was significantly and negatively associated with the individual LI and with the number of RE reports, while an opposite positive relation was observed for LE reports. This finding indicates that an increased gamma-band synchrony goes along with more LE reports and also leads to a reduction of the typical left-lateralization of language. This is in accordance with two prior EEG studies, showing gamma-band oscillations and their synchronization are crucially involved in interhemispheric information transfer during conscious perception of LE syllables^1,21^. It is suggested that synchronized gamma-band activity constitute a fundamental brain mechanism that subserves the cortical computation underlying several cognitive functions^8^, such as auditory perception^58^. As we have also detected an association between functional gamma-band connectivity across bilateral Heschl’s gyri with the dichotic listening performance, we suggest that the vague spatial accuracy of the LORETA approach – which is not better than 1 to 2 cm – spread from pSTG into Heschl’s gyri. The auditory system is partly organized hierarchically and while the Heschl’s gyrus is confined to early sensory analysis of non-speech sounds (e.g. tone), it is particularly the pSTG that is processing and meaningful interpreting a sequence of consonant and vowel speech sounds such as syllables^59^.

Finally, we tested the hypothesis that neural gamma-band synchrony is mediated by the underlying anatomical structure of the IAPs. Although the scatterplots – at least for the pSTG – hint at such an association of stronger pathways and higher gamma-band synchrony, the statistical analyses failed to reach significance. There is evidence from animal studies that sectioning of the corpus callosum leads to a loss of interhemispheric gamma-band synchrony on single-cells^9,60^. In humans, early EEG studies as well as two resting-state fMRI studies reported a striking or complete loss of interhemispheric functional connectivity after callosotomy^61,62^ or callosal agenesis^63,64^. Gamma rhythms are dependent on the activity of fast-spiking parvalbumin-containing gamma-aminobutyric acid-(GABA-)ergic cortical inhibitory interneurons, which in turn are modulated by glutamate N-Methyl-D-Aspartate (NMDA) receptor from excitatory pyramidal cells^65,66^. Thus, the oscillatory phase synchronization is regulated by activity-dependent feedback systems that act to maintain a precise excitatory-to-inhibitory (E/I) balance, which has been shown to play an important role in the development and underspinning of stable perceptions^67^. To date, there is not much literature on relations between EEG-based connectivity and DTI-based connectivity and no mechanistic model exists explaining their mutual interaction^68^. The correlations we found show that both aspects are essentially involved in auditory perception, but there might be a third feature that could explain the remaining gap.

However, there is an essential need to understand the interplay between functional and structural interhemispheric connectivity and language lateralization, because disturbances in any of these components might be of importance in the pathophysiology of auditory phantom perceptions. For instance, neural gamma synchronicity abnormalities, disturbed resting-state fMRI connectivity, as well as altered properties of IAPs have been observed consistently in patients with tinnitus^69–72^ and in patients with AVH in schizophrenia^12–15,73–75^. In line with the present finding, the occurrence and severity of AVH have been related not only to more prominent IAPs^12,14,73^, but also to higher gamma-band synchrony between right and left auditory cortices and a reduced REA during dichotic listening^15,32^. This is of special interest, because even though the majority (80–85%) of right-handed humans exhibit a clear REA^76^, some do not, and in particular those who suffer from AVH in schizophrenia^77–79^. Further, emerging evidence emphasized that abnormal gamma rhythm in AVH patients are due to an E/I imbalance related to glutamate NMDA receptor hypofunction^80,81^. In support, initial 1 H MR spectroscopy studies reported glutamate over-activation in cortical prefrontal and auditory brain areas^82,83^, which in turn goes along with the typical observed neural hyperexcitation of auditory cortices during AVH^84–86^. All these aspects support the notion that both structural and functional interhemispheric auditory connectivity is a relevant contributor to the degree to which language is functionally lateralized and that disturbances in this regard might be related to altered auditory and/or speech processing^87^. In this respect, the combination of multimodal indices – such as EEG-fMRI, DTI and 1 H MR spectroscopy – might be a promising next step to investigate the relation between (1) structural and functional interhemispheric communication, (2) neural and glutamate over-activation within the auditory cortices, (3) interindividual variability of dichotic listening performance (i.e. language laterality), and (4) the emergence and severity of AVH.

A limitation of this study is the limited spatial accuracy of the LORETA approach, although several cross validation studies using simultaneous EEG and fMRI have suggested sufficient validity of the LORETA approach in general^88^. Moreover, a multitude of different measures to quantify functional connectivity are currently available that differ concerning the weighting of phase and amplitude or regarding the removal of zero-phase lag components. We used lagged phase synchronization as this metric removes confounding zero-phase lag contributions, is especially well-established in the auditory domain using dichotic listening tasks^1,15,81^, and commonly used in the clinical field^32,89–91^. However, there might be alternative methods to examine the structure-function relationship, although no metric has emerged as being superior^92^.

In conclusion, the current study shows that the microstructure of individually tracked IAPs connecting homolog pSTG areas as well as interhemispheric gamma-band synchrony between homolog pSTG, are both related to conscious perception of syllables, and further, that higher values within both measures reduce the typical leftward lateralization for language. At the same time, the results show that gamma rhythmic activity is a stronger predictor for perception than the interhemispheric fibre connections. The combination of multimodal neuroimaging approaches opens up new possibilities for understanding the neural correlates of conscious perception.

## Data Availability

The datasets generated during and/or analysed during the current study are available from the corresponding author on reasonable request.
