# Author Correction: The role of functional and structural interhemispheric auditory connectivity for language lateralization - A combined EEG and DTI study

**DOI:** 10.1038/s41598-018-37393-x

**Published:** 2019-02-28

**Authors:** Saskia Steinmann, Rom Amselberg, Bastian Cheng, Götz Thomalla, Andreas K. Engel, Gregor Leicht, Christoph Mulert

**Affiliations:** 1 Psychiatry Neuroimaging Branch, Department of Psychiatry and Psychotherapy, University Medical Center Hamburg-Eppendorf, Hamburg, Germany; 2 Department of Neurology, University Medical Center Hamburg-Eppendorf, 20246 Hamburg, Germany; 3 Department of Neurophysiology and Pathophysiology, University Medical Center Hamburg-Eppendorf, 20246 Hamburg, Germany; 4 Centre for Psychiatry and Psychotherapy, Justus-Liebig-University, Giessen, Germany

Correction to: *Scientific Reports* 10.1038/s41598-018-33586-6, published online 18 October 2018

The Acknowledgements section in this Article is incomplete.

“This research was supported by the DFG (SFB 936 A3/C2/C6 to AKE, GT, CM). We thank Sigrid Boczor for helpful comments on statistical analysis.”

should read:

“This research was supported by the DFG (SFB 936 A3/C2/C6 to AKE, GT, CM). We thank Sigrid Boczor for helpful comments on statistical analysis. Parts of this work were prepared in the context of Rom Amselberg´s dissertation at the Faculty of Medicine, University of Hamburg.”

